# A Mixed-Methods, Randomized Clinical Trial to Examine Feasibility of a Mindfulness-Based Stress Management and Diabetes Risk Reduction Intervention for African Americans with Prediabetes

**DOI:** 10.1155/2019/3962623

**Published:** 2019-08-14

**Authors:** Cheryl L. Woods-Giscombe, Susan A. Gaylord, Yin Li, Carrie E. Brintz, Shrikant I. Bangdiwala, John B. Buse, John D Mann, Chanee Lynch, Pamela Phillips, Sunyata Smith, Karyn Leniek, Laura Young, Saada Al-Barwani, Jeena Yoo, Keturah Faurot

**Affiliations:** ^1^School of Nursing, University of North Carolina at Chapel Hill, Chapel Hill, NC, USA; ^2^Program on Integrative Medicine, Department of Physical Medicine and Rehabilitation, University of North Carolina, Chapel Hill, NC, USA; ^3^Nell Hodgson Woodruff School of Nursing, Emory University, Atlanta, GA, USA; ^4^Department of Biostatistics, Gillings School of Global Public Health, University of North Carolina, Chapel Hill, NC, USA; ^5^Department of Medicine, University of North Carolina School of Medicine, Chapel Hill, NC, USA; ^6^Department of Neurology, University of North Carolina at Chapel Hill School of Medicine, Chapel Hill, NC, USA; ^7^Lehman College, City University of New York, New York, NY, USA; ^8^Health Partners Central Minnesota Clinics, Sartell, MN, USA

## Abstract

African Americans have disproportionately high rates of stress-related conditions, including diabetes and diabetes-related morbidity. Psychological stress may negatively influence engagement in risk-reducing lifestyle changes (physical activity and healthy eating) and stress-related physiology that increase diabetes risk. This study examined the feasibility of conducting a randomized trial comparing a novel mindfulness-based stress management program combined with diabetes risk-reduction education versus a conventional diabetes risk-reduction education program among African American adults with prediabetes and self-reported life stress. Participants were recruited in collaboration with community partners and randomized to the mindfulness-based diabetes risk-reduction education program for prediabetes (MPD; *n* = 38) or the conventional diabetes risk-reduction education program for prediabetes (CPD; *n* = 30). The mindfulness components were adapted from the Mindfulness-based Stress Reduction Program. The diabetes risk-reduction components were adapted from the *Power to Prevent* Program and the *Diabetes Prevention Program*. Groups met for eight weeks for 2.5 hours, with a half-day retreat and six-monthly boosters. *Mixed-methods strategies were used to assess feasibility*. Psychological, behavioral, and metabolic data were collected before the intervention and at three and six months postintervention to examine within-group change and feasibility of collecting such data in future clinical efficacy research. Participants reported acceptability, credibility, and cultural relevance of the intervention components. Enrollment of eligible participants (79%), intervention session attendance (76.5%), retention (90%), and postintervention data collection attendance (83%, 82%, and 78%, respectively) demonstrated feasibility, and qualitative data provided information to further enhance feasibility in future studies. Both groups exhibited an A1C reduction. MPD participants had reductions in perceived stress, BMI, calorie, carbohydrate and fat intake, and increases in spiritual well-being. Considering the high prevalence of diabetes and diabetes-related complications in African Americans, these novel findings provide promising guidance to develop a larger trial powered to examine efficacy of a mindfulness-based stress management and diabetes risk-reduction education program for African Americans with prediabetes.

## 1. Introduction

Approximately 86 million US adults (37%) have prediabetes [1]. Prediabetes is characterized by blood glucose levels above normal but below the criteria for a diagnosis of diabetes [1]. Most people with prediabetes are expected to be diagnosed with type 2 diabetes within 10 years [2]. Preventive efforts are critical; diabetes is a leading cause of death and disability in the US, with yearly direct (medical) and indirect (disability, work loss, and premature mortality) costs estimated at $245 billion [1].

African Americans are disproportionately affected by diabetes and diabetes-related morbidity [1, 3]. Compared to White Americans, African Americans are almost twice as likely to have diabetes [1, 4] and more than twice as likely to suffer from diabetes-related morbidities (blindness, lower limb amputations, and kidney disease) [3]. Hence, preventing diabetes among African Americans has become a primary focus of the National Institutes of Health Strategic Plan on Minority Health Disparities [5].

Prevention strategies (e.g., physical activity and a 5–10% weight loss) can significantly delay the onset or reduce the development of diabetes [2, 6]. Although those who increase physical activity and improve their diets have reduced risk of developing diabetes [7], initiating and sustaining the necessary behavioral changes are difficult. Notably, African Americans and particularly African American women were the least successful at losing weight compared with men and women of other racial/ethnic groups in the lifestyle intervention arm of the *Diabetes Prevention Program* (DPP) study [8]. Similar results for African American women were found in real-world translation studies of the DPP in high-risk individuals, suggesting a need for enhancing evidence-based interventions to address potential barriers to lifestyle change in this population [9, 10].

Individuals who experience higher stress have greater barriers to making healthy lifestyle changes, including decreased engagement in healthy behaviors (e.g., exercise) and increased unhealthy lifestyle behaviors (e.g., excessive consumptions of food, alcohol, or nicotine) [11–13]. Such lifestyle changes are often advised by healthcare providers to lower the risk for chronic illnesses such as diabetes. One study found that increased psychological stress was a major roadblock to weight control in African American women [14]. In addition to the negative impact of stress on implementation and management of lifestyle changes, physiological responses to stress, in particular hypothalamic-pituitary-adrenal- (HPA) axis activation and dysregulation may have a direct negative impact on insulin resistance and glucose metabolism [15].

African Americans have high exposure to a broad range of psychological stressors and are particularly at risk for stress-related health conditions [11]. Theories that seek to explain stress-related disparities include allostatic load [16] and the weathering framework [17, 18]. Allostatic load places emphasis on the impact of cumulative risk that results from chronic exposure to life challenges and stress [16]. According to Geronimus, “the cumulative impact of repeated experience with social, economic, or political exclusion” is a stressor responsible for current health disparities (p. 133) [18]. Excessive demands on regulatory systems compromise cardiovascular, autonomic, metabolic, and neuroendocrine activity, leading to disease states such as diabetes [19]. In addition, research on the Environmental Affordances Model [20] suggests that African Americans might engage in unhealthy behaviors as a coping strategy in response to chronic stress, instead of making recommended lifestyle changes. While stress exposure may not always be controllable, stress-management interventions may alter the response to stress and decrease diabetes risk in individuals with metabolic risk factors [21–26], including prediabetes.

Mindfulness-based stress reduction (MBSR) is an evidence-based stress management approach involving training in the intentional self-regulation of attention. Techniques involve learning to place awareness on present-moment experiences and letting go of fixation on thoughts of past and future [27]. Mindfulness training has been used in randomized controlled clinical trials as an effective intervention for reducing stress and increasing well-being in a variety of healthy and clinical populations [28, 29]. In particular, mindfulness training has had positive outcomes in individuals with type 2 diabetes and those who are overweight or at risk for metabolic illness [22–26, 30, 31].

**Figure 1:**
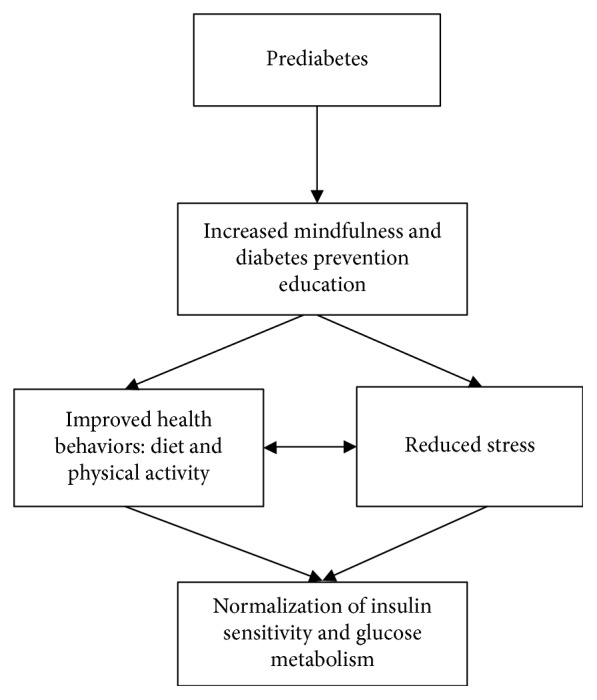
Conceptual model.

A growing body of research suggests that mindfulness training and other types of mind-body or meditation training may be culturally relevant interventions for stress-related conditions in African Americans [21–23, 32–35]. Previous studies have successfully incorporated mind-body and other stress reduction techniques in lifestyle programs to promote weight loss in African American women [21, 22]. However, there remains a need for well-controlled research on the feasibility of a more comprehensive mindfulness-based diabetes prevention program specifically for African Americans with prediabetes, to determine whether stress reduction can promote diabetes risk reduction, and whether African Americans in particular might benefit from interventions that improve stress-related processes and enhance engagement in healthy behaviors critical for preventing diabetes [21]. Thus, we designed a pilot trial to examine the feasibility of implementing an eight-week mindfulness-based diabetes risk-reduction education program for prediabetes (MPD) versus a conventional diabetes risk-reduction education program for prediabetes (CPD) among African American adults with prediabetes who are experiencing stress. The MPD curriculum included an adapted mindfulness-based stress reduction training component as well as conventional diabetes risk-reduction education, while the CPD included only conventional diabetes risk-reduction education. Our goal was to examine the feasibility of implementing an intervention to determine whether the MPD approach would reduce risk of developing diabetes by reducing stress levels as well as by facilitating positive health behaviors (e.g., changes in diet and physical activity) (conceptual model in Figure 1). Key aspects of the study design included: (1) feasibility (recruitment, attendance at intervention and data collection sessions, group cohesion, retention, and intervention credibility) and (2) examining within-group changes (metabolic, physiological, psychological, and health behavior variables relevant to diabetes risk). We did not examine between-group differences in outcomes, as this was a feasibility trial not powered to provide reliable estimates of intervention efficacy.

## 2. Materials and Methods

Quantitative and qualitative methods were utilized to assess the feasibility of implementing a pilot sequential mixed-methods, two-arm, randomized controlled trial to compare two group-based interventions—a mindfulness-based diabetes risk-reduction education program (MPD) vs. a conventional diabetes risk-reduction education program (CPD). Materials and methods for the quantitative and qualitative components are described below. Prior to the initiation of the study, procedures and informed consent forms were reviewed and approved by the University of North Carolina Institutional Review Board.

### 2.1. Quantitative Methodology

The overall purpose of the quantitative portion of the study was to assess feasibility of recruiting and engaging a group of African American adults with prediabetes who were willing to enroll in the study and attend intervention, booster, and data collection sessions. Feasibility of intervention implementation, intervention credibility, and data collection methods was also assessed.

### 2.2. Recruitment, Screening, and Enrollment

This trial was designed to have a three-step screening and recruitment process prior to enrollment and randomization into the two intervention arms. To reduce prerandomization drop out, study subjects were recruited in cohorts. Study procedures and consents were reviewed and completed with participants at each stage of the screening process. At Screening Step 1, individuals who were identified as African American adults, aged 25 or older, were recruited from healthcare agencies and community screening events (e.g., community organizational events, health fairs, and churches). To determine the risk for prediabetes, a Diabetes Risk Questionnaire (DRQ) was administered, a modified version of the questionnaire developed for the National Diabetes Education Program (Table 1) [36].

At Screening Step 2, participants with a DRQ score >10 were screened for inclusion and exclusion criteria (Table 2). At Screening Step 3, interested, eligible participants visited the UNC Clinical and Translational Research Center (CTRC), where they provided written informed consent and completed fasting laboratory testing and baseline questionnaires. This final screening step helped to confirm the prediabetes status of potential participants (versus a normal or diabetes glucose level). During this final three-hour screen visit, participants were determined to be eligible if they met criteria for prediabetes by either one of these measures: fasting blood glucose (100–124 mg/dL; 5.6–6.9 mmol/L), an oral glucose tolerance test (OGTT) (140 mg/dl–199 mg/dl), or glycosylated hemoglobin A1C levels (5.7–6.4%; 39–46 mmol/mol). All participants were encouraged to follow-up with their health providers after this screening step. For those who were found to have glucose levels in the diabetes range, the study physician was notified and permission was obtained to inform the participants' healthcare providers for additional follow-up steps. Participants were informed that if they were eligible for the study and agreed to participate they would receive remuneration for parking, travel, and payment for attendance at each data collection laboratory visit. In addition, they received gifts at intervention sessions, including water bottles, lunch bags, reusable grocery shopping bags, an exercise mat, and a notebook and pen.

### 2.3. Randomization

Eligible individuals with laboratory results in the prediabetes range were then invited to participate in the “We Can Prevent Diabetes” program (described below). A random number generator program was used to randomize participants and to ensure equal numbers of MPD and CPD assignments within a permuted variable block size of 4–8 subjects. This system used sequential sealed envelopes to ensure allocation concealment. The study coordinator chose each envelope by a sequential study identification number and notified each individual of their allocation the day before the classes were to begin.

### 2.4. Blinding

Although the nature of the interventions did not allow for blinding of the instructors or participants, steps were taken to minimize differences in participant expectancy, as follows: the experimental interventions were advertised under the umbrella title of “We Can Prevent Diabetes” and described to participants as two group-based diabetes prevention educational interventions, both of which may lower blood glucose. No study literature identified either group as more efficacious. To assess whether participants assigned to the CPD had the same expectation of benefit as those assigned to the MPD, all participants completed a credibility assessment [38] after the first and seventh intervention sessions. The statistician and data manager were blinded with respect to group assignment.

### 2.5. Interventions

Once randomized, participants attended one of the two eight-week programs (MPD or CPD). Each weekly session lasted for 2.5 hours, in addition to a four-hour half-day retreat held on a Saturday, plus six 1.5-hour booster sessions at one-month intervals following the eight-week program. All MPD and CPD class sessions took place at a local public school, during times when school was not in session.

Both MPD and CPD groups included a weekly 30-minute diabetes risk-reduction education segment facilitated by a certified diabetes educator. This segment included information and discussion based on topics from (1) an abbreviated version of the landmark *Diabetes Prevention Program* content, developed by the Centers for Disease Control and Prevention (CDC) [39]; (2) *Power to Prevent,* a culturally tailored diabetes prevention program for African Americans developed by the National Diabetes Education Program (NDEP), a collaborative effort including the CDC and the National Institutes of Health [40]; (3) diabetes patient education videos produced by the American Association of Diabetes Educators [41]; (4) diabetes risk-reduction materials produced by the CDC [42, 43]; and (5) content from a previously funded project conducted by the investigators: the Integrative Diabetes Prevention and Self-Management Program [44]. The diabetes educator presented information about food choices, shopping, cooking, and dining out; weight control; physical activity guidelines; emotional aspects of diabetes; and changing eating behaviors. Both groups also received a healthy snack. Participants in both groups were also asked to keep and turn in weekly logs of healthy behaviors that had been encouraged during class sessions, including physical activities and diet. For both the MPD and CPD groups, the six-monthly booster sessions reviewed topics covered in their respective programs and provided an opportunity for participants to receive support for sustained behavioral change.

#### 2.5.1. MPD Intervention

The overall goals of the MPD intervention were to provide participants with diabetes risk-reduction education (as in the CPD intervention) as well as training in mindfulness-based stress management that was adapted to include information and behavioral-change content to promote diabetes risk reduction for African Americans with prediabetes. The first one-hour segment of each class was taught by an experienced Mindfulness instructor who was also knowledgeable about diabetes prevention. The mindfulness-based stress management segment of the MPD program followed the basic format outlined in the Kabat-Zinn's MBSR program but was adapted by shortening the amount of training time to one hour and including discussions on relevance to diabetes risk reduction. The MPD participants learned mindfulness-based stress management concepts, skills, and practices. This segment was followed by a one-hour diabetes risk-reduction education segment, facilitated by a certified diabetes educator, which included a question and answer session and discussions about barriers to and facilitators of behavior-change strategies. Time during each session also included discussions on the previous week's homework, strategies for practically applying the lesson of the week at home, a break for healthy snacks, and two bathroom breaks. Homework assignments included mindfulness skill practices that had been taught during the mindfulness training segment (e.g., mindful breathing, body scan, breathing space, mindful movement, and mindfulness in everyday life) and diabetes-prevention activities adapted from the NDEP's *Power to Prevent* lifestyle change program materials [40]. MPD participants were asked to record their personal experiences of the mindfulness practices, as well as diabetes risk-reduction applications, including perceived barriers to and facilitators of behavioral change. Booster sessions involved review and practice of mindfulness skills, review of strategies for overcoming barriers and sustaining lifestyle behavior changes to reduce risk factors for diabetes, and discussion of participants' successes and challenges in implementing the program.

#### 2.5.2. CPD Control Intervention

The CPD attention-control group provided experiences that were similar in time to the MPD, including amount of diabetes-prevention educational content, amount of regular participant contact with instructors, and amount of homework assigned. However, instead of content on mindfulness for one hour of the session, the CPD participants engaged in activities and games directly related to the content delivered by the CPD facilitators. As in the MPD group, there was a one-hour diabetes risk-reduction education segment, facilitated by a certified diabetes educator, which included a question and answer session and discussions about barriers to and facilitators of behavior-change strategies. As in the MPD group, there was a break for healthy snacks and two bathroom breaks. The CPD was facilitated by health educators experienced in working with small groups, knowledgeable about diabetes prevention, and trained to involve participants in discussion of diabetes risk reduction [37, 40]. CPD homework assignments and booster sessions matched those of the MPD group in terms of time commitment and relevance to diabetes prevention.

### 2.6. Protocol Fidelity

All intervention sessions were audiotaped, and a research team member attended each group session to conduct fidelity monitoring. Protocol fidelity was discussed at team meetings, and deviations were addressed promptly.

### 2.7. Quantitative Measures

Unless otherwise specified, administration of outcome and process measures took place upon entry into the study, within two weeks after completion of the eight-week programs and again at three and six months after intervention. A demographic questionnaire was completed at intake, collecting each participant's age, marital status, years of education, work status, and approximate family income.

#### 2.7.1. Quantitative Feasibility Measures

Quantitative feasibility measures included (1) recruitment, (2) attendance at intervention sessions and data collection attendance, (3) group cohesion, (4) retention of participants in the study, and (5) credibility.

*(1) Recruitment*. Aspects of the recruitment, screening, and enrollment process are key indicators of study feasibility. As described previously, this study incorporated a multistep process to screen widely for potential participants with elevated risk for prediabetes, determine whether potential participants meet eligibility criteria for definitive laboratory testing, meet criteria for prediabetes, and meet final criteria for randomization to one of the two intervention groups. Recruitment numbers and participation rates among those invited can provide important information for the design of future studies.

*(2) Attendance*. Participant adherence and engagement were measured in two ways: by assessing (1) intervention session attendance and (2) data collection attendance at clinical laboratory visits.

*(3) Group Cohesion*. The Group Cohesion Scale [45] assesses participants' perceived trust, compatibility with, and commitment to the group and was measured to assess whether these specific group characteristics were different between the groups. The Group Cohesion Scale was administered during Sessions 2 and 7. It consists of 12 questions rated on a scale from 1 = *strongly agree* to 7 = *strongly disagree*. A sample item is “I am willing to work at not missing sessions.” Items were reverse-coded prior to scoring so that scores ranged from 12 to 84, with higher scores indicating higher group cohesion.

*(4) Retention*. Retention was measured by assessing the percentage of participants retained in the study through the final follow-up assessment period (6-month follow-up visit).

*(5) Credibility*. An additional question regarding feasibility involved the extent to which either intervention was credible for this sample. The Credibility Scale was adapted from an instrument developed by Borkovec and Nau [38] that measured the participant's expectation of benefit from a particular treatment, once it had been described. Originally designed to test the credibility of standard psychotherapy vs. placebo treatments, it has been successfully modified to assess credibility and expectation of benefit of various interventions and conditions [46, 47]. For this study, the scale was revised by rewording some of the items to ask specifically about the credibility and expectation of benefit of the MPD and CPD interventions for the prevention of diabetes. The instrument was administered at the beginning of Session 2 and Session 7 of the interventions. The response options ranged from 0 = *not at all* to 9 = very, with higher ratings indicative of higher credibility. Participants rated how logical the intervention was for them, how confident they were that the intervention would help them prevent diabetes, how confident they would be in recommending a friend to engage in the intervention, how important they think it is that the intervention be offered to others, and how successful they believe the intervention will be in decreasing other problems such as tension, anxiety, or insomnia.

#### 2.7.2. Quantitative Outcome Measures

Quantitative outcome measures included (1) diabetes biomarkers, (2) body composition measurements, (3) perceived stress, (4) quality of life, and (5) diet and activity measures.

*(1) Diabetes Biomarkers*. The homeostasis model assessment of the insulin resistance (HOMA-IR) value (a measure of insulin resistance) was calculated from fasting insulin and fasting glucose values to assess preliminary efficacy of the interventions. HOMA-IR models have been employed in over 500 published studies, [48] including behavioral intervention studies with persons at risk for diabetes [49–52] and a mindfulness intervention in obese individuals with binge eating disorder [52]. Glycosylated hemoglobin levels (A1C) were also obtained at baseline, three months postintervention, and six months postintervention, to evaluate glucose control over time.

*(2) Body Composition Measurements*. Body weight was assessed on a digital scale (to the nearest 0.1 kg) that is accurate within ±0.05%. Body height was measured to the nearest cm using an adult stadiometer. Body mass index (BMI) was calculated as weight (kg) divided by height (m^2^). BMI and waist circumference are both valid measures of obesity [53, 54] and have been shown to be predictors of metabolic and cardiovascular risk [55, 56]. Waist circumference was measured twice at the midpoint between the upper iliac crest and lower costal margin in the midaxillary line for males and females [57]. Hip circumference was measured twice at the maximum width of the buttocks or gluteofemoral fold. The waist-to-hip ratio (WHR) was calculated as the mean waist circumference divided by the mean hip circumference [57].

*(3) Perceived Stress*. The Perceived Stress Scale-14 (PSS-14) measures self-appraised stress during the last month and was administered only at the screening visit to assess the degree to which respondents found their lives unpredictable, uncontrollable, and overloaded [58]. Response options range from 0 = *never* to 4 = *very often*. Scores range from 0 to 56, with higher scores indicative of greater perceived stress. The PSS-4, with scores ranging from 0 to 16, is an abbreviated version of the PSS-14 [58] and was administered at baseline and two weeks, three months, and six months postintervention. The PSS-4 has yielded good psychometric properties for African Americans, with a mean score of 4.6 [59].

*(4) Quality of Life*. The Functional Assessment of Chronic Illness Therapy, Spiritual Well-Being Expanded Version Scale (FACIT-Sp-Ex), is a component of the comprehensive FACIT Measurement System, which includes scales to measure generic and disease-specific health-related quality of life [60]. The 23-item scale assesses sense of meaning, peacefulness, faith, and relational aspects of well-being and has demonstrated good psychometric properties [61, 62].

*(5) Diet and Activity Measures*. The Seven-Day Physical Activity Recall [63] assesses the frequency, duration, type, and perceived intensity of all physical activities in the previous week. Based on the questionnaire, energy expenditure (kilocalories) was assessed [64]. The questionnaire has good test-retest reliability and validity compared with accelerometer readings [65] and has been used successfully in a study of African American adults and children with diabetes [64].

The Fred Hutchinson Cancer Research Center Food Frequency Questionnaire (FHCRC-FFQ) was adapted from the Block Food Frequency Questionnaire by Kristal and colleagues [66, 67]. The measure has been adapted to ethnic dietary habits and has been validated with minority populations [66, 68]. Outcomes analyzed included daily intake of calories, fat, and carbohydrates. Although other dietary intake variables were measured (e.g., vitamin D and protein), they are not reported for purposes of this paper.

### 2.8. Qualitative Study Methodology

The overall purpose of the postintervention qualitative portion of the study was to assess acceptability of the intervention by (a) identifying the aspects of the program that were most useful and challenging, so as to make any needed program modifications; (b) exploring how practices taught in this program were congruent with, or conflicted with, cultural and or spiritual beliefs or practices; and (c) learning about recommendations for specific changes that could enhance recruitment and retention. At the time of consent into the study, permission was requested to conduct recorded in-depth interviews. After intervention, in-depth interview techniques were used to explore attitudes and experiences with mindfulness among participants in the MPD group. These approaches facilitated the identification of emergent themes and their impact on program participation and satisfaction and were particularly useful for exploring the participants' perspectives regarding the intervention content and for gaining understanding of and synthesis with the quantitative data. Questions were open-ended and probing (Table 3), with all respondents receiving the same questions in approximately the same order. Respondents were encouraged to answer in their own words, elaborate on their responses, explain the meaning of terms employed, and discuss meanings they gave to their experiences.

### 2.9. Analytic Plan

#### 2.9.1. Quantitative Analysis

Based on the study design, unobserved outcome data were assumed to be missing at random (MAR). All randomized participants were included for intention to treat (ITT), using two strategies for managing missing data. For the psychological outcomes with <5% missing data, we used multiple imputations. For all data with >5% missing values, we applied the last value carried forward and the next value carried back for ITT. Confidence intervals were used to examine the within-group changes. All analyses were conducted using SAS software version 9.3 [69]. Although the current study's primary objective was to investigate intervention feasibility and it was not powered to examine efficacy, we conducted analyses to identify patterns of the within-group change in metabolic variables, stress, health behaviors, and quality of life (i.e., spiritual well-being) in the two intervention arms to inform the design and development of a larger, randomized controlled-trial powered to detect efficacy.

#### 2.9.2. Qualitative Analysis

Interview data were analyzed in accordance with applied thematic analysis [70]. Strategies were used to enhance the credibility, trustworthiness, and rigor of the analysis process and results [70]. First, five collaborators (CG, SG, KF, CL, and PP) who were involved with the design and implementation of the four-year study identified and discussed patterns found in the data and produced preliminary codes. Next, two research team members not involved with the design or implementation of the study (JY and SA) coded the data based on the research questions. Because this was their first encounter with these data, they brought a fresh perspective to the analysis, enhancing credibility and dependability of the findings. After independent coding, they compared codes within and across interviews and reached agreement on any differences. Finally, the codes were reviewed with original team members. All participated in summarizing the findings.

## 3. Results

### 3.1. Recruitment and Screening

**Figure 2:**
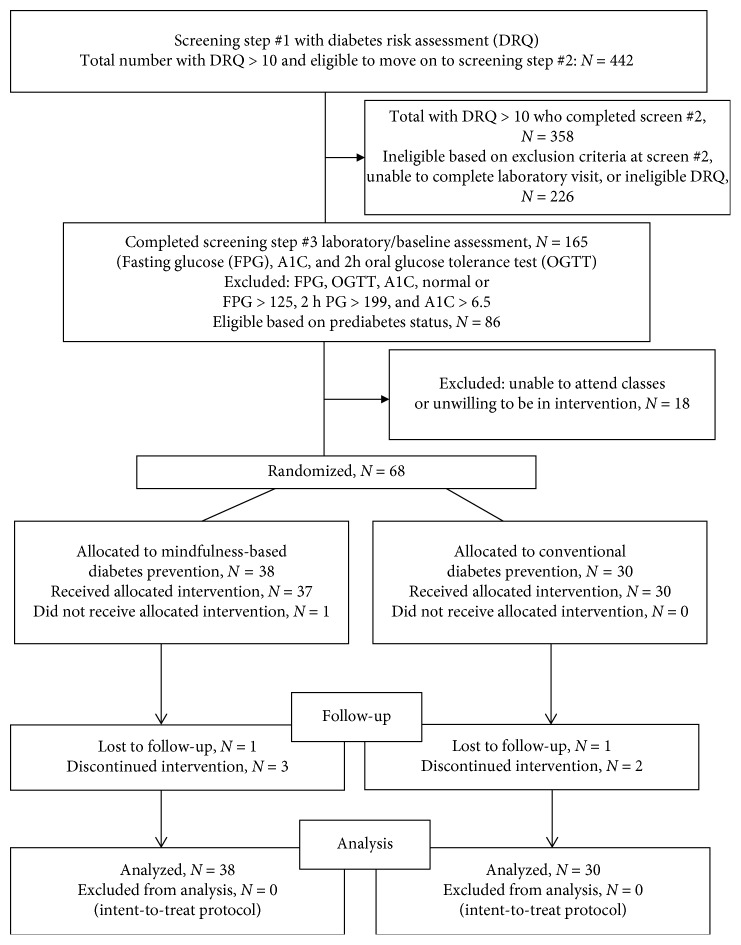
CONSORT flow diagram.

Using the Diabetes Risk Questionnaire (DRQ), 442 persons were screened to identify individuals with elevated diabetes risk factors (DRQ score >10). Among the individuals screened, 81% exhibited risk factors for prediabetes (*n* = 358). Among those who passed the initial stage of screening, 46% (*n* = 165) completed laboratory testing to identify or confirm prediabetes status and to complete the baseline survey assessment. Of those who completed laboratory testing, 52% (*n* = 86) were diagnosed with prediabetes (OGTT between 140 and 199 mg/dl, A1C between 5.7 and 6.4%, and/or fasting glucose of 100–125 mg/dl). Among those with prediabetes, 41% (*n* = 68) met all other eligibility criteria for enrollment and randomization to either the MPD or the CPD intervention groups (Figure 2).

### 3.2. Participant Characteristics

Demographic characteristics of the CPD and MPD groups were similar (Table 4). The sample included African American participants with generally high education and income. Participants ranged in age from 29 to 70 years (MPD: *M* = 52.66, SD = 9.94; CPD: *M* = 52.48, SD = 9.55).

### 3.3. Attendance

#### 3.3.1. Intervention Session Attendance and Group Cohesion

Good intervention session attendance was defined for this study as attendance of at least six or more of the eight intervention sessions. On average, 76.5% of all participants had good attendance (76.3% for the MPD group and 76.7% for the CPD group).

#### 3.3.2. Data Collection Attendance

A major part of accessing feasibility included monitoring the extent to which participants returned to the data collection site at the UNC CTRC for data collection at the postintervention follow-up sessions. Eighty-three percent of participants attended the 2-week postintervention data collection session. Eighty-two percent of participants attended the 3-month follow-up data collection session, and 78% of participants attended the 6-month follow-up data collection session.

### 3.4. Group Cohesion

Scores for the Group Cohesion Scale, assessed during Session 2 and Session 7 and were moderately high for both groups (MPD group: *M* = 59.33, SD = 14.74, to *M* = 67.36, SD = 5.90, respectively; CPD group: *M* = 69.29, SD = 11.92, to *M* = 67.76, SD = 15.12, respectively), with no between-group differences by Session 7.

### 3.5. Retention

Of the 68 participants randomized, 90% were retained. A total of seven participants were not retained in the study. Two participants were lost to follow-up (*n* = 1 in MPD and *n* = 1 in CPD; these two participants were not reachable by telephone, email, or postal mail). Five participants requested to discontinue intervention attendance due to personal reasons (*n* = 3 in MPD and *n* = 2 in CPD; reasons included work schedule constraints, death, or illness in their family).

### 3.6. Credibility

After the first and seventh sessions, both groups identified the interventions as credible, based on their responses to the credibility questionnaire, with the CPD seen as more credible (Table 5). Both groups described the interventions as logical, felt confident that the interventions could help them reduce risk for diabetes, would recommend the intervention to others, thought that it would be important to make the intervention available to others with prediabetes, and felt that the interventions could help with psychological concerns such as tension, anxiety, and insomnia.

### 3.7. Acceptability

Participants reported no adverse effects from participating in the intervention. Postintervention interviews were conducted with participants in the MPD (*N* = 23) and CPD (*N* = 25). Individuals in both groups uniformly reported increased knowledge about strategies to improve health, including diet and exercise. In addition, all individuals in both groups reported changing their behaviors as a result of the interventions. Specifically, participants reported increasing their knowledge about and behaviors related to reading of labels, reducing portion sizes, choosing healthier snacks, reducing sugar, increasing vegetable intake, incorporating new exercises, and reducing fat intake (reduced “greasy” foods), calories, and carbohydrates. In addition, most participants in the MPD group mentioned using mindfulness, breathing techniques, and conscious eating behaviors and stated that they were more aware of stress processes and the need for stress reduction.

No participants in either group disclosed any incongruence of the intervention components with cultural or spiritual beliefs. All MPD participants expressed feelings of satisfaction with the mindfulness component of the intervention. One participant expressed that she enjoyed meditating and being quiet and connected it to her Baptist faith, noting that the mindfulness practices enhanced her spiritual practice. Other participants also noted the benefits of mindfulness for stress management and awareness of health behaviors. No participants expressed dissatisfaction with learning about mindfulness as part of diabetes risk-reduction education.

Several participants shared information that will help to improve the design of a larger trial. Participants described a number of challenges to attending the intervention and booster sessions, including work conflicts, family obligations, and tiredness after work. Participants' suggestions for addressing these barriers included scheduling sessions on weekends or later in the evenings. It is interesting to note that we offered the first cohort on a Saturday morning, which had the lowest attendance rates. The remaining four cohorts were held on weeknights beginning around 6:30 pm. Some participants also suggested avoiding scheduling sessions on Tuesday or Thursday nights (sports-game nights for children), having sessions at a more centrally located community or church setting, and having longer sessions with fewer total session days. In contrast to those who suggested fewer session days, some participants requested more face-to-face sessions, twice-monthly booster sessions, additional half-day sessions, and a longer follow-up period beyond six months. Others verbalized appreciation for having sessions at a school in the target community versus at the medical center.

Some participants expressed challenges to their attendance at the UNC CTRC hospital-based laboratory data collection visits including traffic, parking, and medical center location. Individuals' suggestions for overcoming these challenges included having nighttime appointments and an off-site location, such as a local community clinic. Several participants also reported challenges with “making time” to meditate and do the homework. Some participants noted challenges with balancing work and family obligations to meditate consistently, cook and eat healthier foods, and integrate physical activity into their lives. Individual participants also made suggestions regarding the need for an increased group and peer support, enhanced accountability, access to study-specific videos to enhance exercise, and tips for organizing time to incorporate exercise and healthy behavioral change.

### 3.8. Quantitative Outcome Results

Although the current study's primary objective was to investigate intervention feasibility and it was not powered to examine efficacy, we conducted analyses to identify patterns of within-group change in metabolic variables, stress, health behaviors, and quality of life (i.e., spiritual well-being) in the two intervention arms to inform the design and development of a larger, randomized controlled-trial powered to detect efficacy. All results are reported in Table 6. Both the MPD and CPD groups experienced significant reductions in A1C from baseline at 3 months and 6 months follow-up. Only the MPD group experienced significant reductions in BMI at 3 months. In addition, the MPD group experienced significant reductions in perceived stress at 3 months follow-up, whereas there were no significant changes in perceived stress in the CPD group. The MPD group experienced reductions in daily calorie and carbohydrate intake at 3 and 6 months and reductions in daily fat intake at 6 months. Overall, the quantitative results did not reveal significant quantitative changes in health behaviors among the CPD group. The MPD group experienced significant increases in total spiritual well-being at 6 months, while the CPD group experienced significant decreases in spiritual well-being at 6 months. There were no significant changes in HOMA-IR, cortisol, waist-to-hip ratio, or physical activity levels in either group.

## 4. Discussion

This is the first study to investigate the feasibility of implementing a comprehensive mindfulness-based diabetes risk-reduction education program, as compared to a conventional diabetes risk-reduction education program, for African American men and women with prediabetes. This study builds on other findings supporting the potential of mind-body interventions for improving well-being in African American adults with elevated risk for metabolic illness [21–24] Other than our own research, there has been only one previous research study that implemented formal mindfulness training to promote reduction in metabolic risk in African American adults [24]. This single-group intervention focused on weight loss with mindful eating in African American women following their treatment for breast cancer (*n* = 22). After six biweekly, 120-minute mindfulness group sessions and individual sessions with a registered dietician over a 12-week period, followed by six-biweekly supportive telephone calls with study staff over a 12-week period, participants experienced statistically significant improvements in mindful eating and weight loss over time (at 13 weeks and at the end of the 24-week study). Other previous studies with women participants only have included lifestyle education sessions involving brief or informal, mind-body training segments (e.g., relaxation techniques, guided imagery, diaphragmatic breathing, and/or mindfulness), incorporated into interventions designed to promote healthy eating, physical activity, or weight loss [21–24]. Yet, none have included a more traditional eight-week mindfulness-based stress management curriculum integrated with diabetes risk-reduction education.

Considering the higher prevalence of diabetes and diabetes-related complications in African Americans compared with White Americans, the findings provide novel and important information for guiding and improving future research and informing diabetes prevention efforts in African Americans. Positive attitudes and experiences related to mindfulness training were expressed among our sample of African American adults with prediabetes. Participants described the intervention as acceptable and culturally relevant. It is interesting to note that although both groups considered their intervention to be credible, the participants in the conventional diabetes risk-reduction group (CPD) provided slightly higher overall ratings of program credibility. In a future, definitive RCT, an orientation to the overall program curriculum, can be included during the first session with the intentional content to enhance equitable and high perceptions of credibility among both intervention arms.

Participants in both groups voiced general approval regarding the components of their interventions and reported increases in knowledge and changes in behavior because of engagement in the study. Participants in the MPD group, but not the CPD group, reported incorporation of mindfulness techniques, including increased awareness of stress and increased consciousness of eating mindfully.

The qualitative findings were congruent with and provided insights into the pattern of results. In the quantitative findings, both groups experienced reductions in A1C. There was a decrease in perceived stress among MPD participants, but not CPD participants, at three months postintervention and an increase in spiritual well-being in the MPD group at 6 months. In addition, changes in dietary habits were noted only in the MPD group. The patterns of reduction in perceived stress in the MPD group, but not the CPD group, suggest a potential pathway for improved dietary habits among MPD participants. Qualitative and quantitative findings of increased stress management, decreased stress, and increased ability to engage successfully in lifestyle changes are plausible pathways towards ultimately reducing diabetes risk.

When considered with this growing body of research evidence in support of mind-body interventions for risk reduction of metabolic conditions, the mindfulness-based diabetes risk-reduction education program implemented in this study holds promise, giving its feasibility, acceptability, and impact on stress reduction, quality of life, and improved dietary intake. Nonetheless, this study had limitations worth noting and addressing in future research. Although payment for public transportation, mileage, and parking and other financial incentives were provided for all study participants to reduce barriers to participation (e.g., study visits at a major medical center and multiple educational intervention visits), some participants expressed that the location of the assessments, the amount of time needed for assessments, and the intervention itself were challenges to participation. Therefore, although the sample was socioeconomically diverse, it may be that these challenges contributed to overrepresentation of individuals with higher than average education and income. This was also the case in the other three studies that implemented mind-body strategies to reduce metabolic risk factors in African American adults [21, 22, 24].

Overall, participants discussed suggestions for improvements in timing of intervention sessions, location of intervention sessions, and data collection sites and strategies needed to support health behavior change and maintenance at home. A future, definitive clinical trial can be designed to address these concerns (Table 7). In our study, follow-up qualitative data of participants suggested that family, professional, and other competing demands created challenges to engagement in the study as designed and to incorporating diet, exercise, and stress management changes in their lives. Enhanced strategies to make study components accessible may enhance socioeconomic diversity and study engagement among participants in follow-up research. Suggestions offered by participants included providing aids and prompts to enhance mindfulness meditation at home, offering guidance on time management, incorporating a buddy system to enhance accountability in adhering to program activities, and scheduling study visits in community locations closer to the homes of participants. The incorporation of a community advisory board during the development and implementation of the next phase of research also can be valuable for maximizing cultural relevance, acceptability, outreach/recruitment, impact, and future translation and dissemination of the project.

## 5. Conclusions

In summary, this study provides data to support feasibility for future implementation of a larger trial of a mindfulness-based diabetes prevention program for African Americans. Findings will help to guide future research to determine if instruction in mindfulness, an evidence-based stress management skillset, is efficacious in improving insulin and glucose metabolism when integrated with standard diabetes education. Such future research might also facilitate advanced understanding of mechanisms of action involved with mindfulness interventions. In addition, with larger samples, subgroup analyses can be performed (e.g., by demographics) to identify subjects who respond most positively to mindfulness-based stress reduction and diabetes risk-reduction intervention components. The integrated, quantitative, and qualitative findings provide rich foundational information for developing a larger, randomized controlled clinical trial that is adequately powered to investigate the efficacy of a mindfulness-based diabetes risk-reduction education program compared to a conventional diabetes risk-reduction education program in producing sustainable, stress-reducing, and health-enhancing behavioral changes among African American adults with elevated risk for diabetes. Flexible class dates and make-up sessions to fit participants' busy schedules, as well as an accessible community site for data collection (e.g., laboratory, survey, and qualitative data) and intervention implementation, are major factors that will improve study feasibility for a future trial.

## Figures and Tables

**Table 1:** Diabetes Risk Questionnaire (DRQ): Screening Step 1.

Question	Yes	No
Are you a woman who has had a baby weighing more than 9 pounds at birth?	1	0
Do you have a sister or brother with diabetes?	1	0
Do you have a parent with diabetes?	1	0
Have you been told that you have high blood pressure?	2	0
Have you been told that your cholesterol (lipid) levels are abnormal?	2	0
Find your height on the chart.^^*∗*^^		
Do you weigh as much as or more than the weight listed for your height?	5	0
Are you under 65 years old and get little or no exercise in a typical day?	5	0
Are you between 45 and 64 years old?	5	0
Do you have an African-American or Hispanic or American Indian family background?	5	0
Are you 65 years old or older?	9	0

Notes: Participants were instructed to add the number of points listed for each “Yes” answer, and speak with study staff if the score was 10 or greater. ^^*∗*^^Participants were provided with a body mass index chart with a list of heights and corresponding weights meeting overweight status.

**Table 2:** Inclusion and exclusion criteria for intervention.

*Inclusion criteria*
(1) African American
(2) Aged 25 and 65 years
(3) Meets ADA criteria for prediabetes either by hemoglobin A1C of 5.7–6.4% (39–46 mmol/mol), fasting plasma glucose (FPG) of 100–124 mg/dl (5.6–6.9 mmol/L), or glucose of 140–199 mg/dl (7.8–11.1 mmol/L) at 2 hours in an oral glucose tolerance test (OGTT) at Clinical Trials Research Center visit
(4) Perceived stress Scale-14 [37] score >5 or self-report of at least “some” general life stress^a^

*Exclusion criteria*
(1) Diabetes diagnosed by a healthcare provider
(2) Past or current use of hypoglycemic medication (except for gestational diabetes)
(3) Disease associated with disordered glucose metabolism (e.g., Cushing's Syndrome)
(4) Regular use of medications associated with impaired glucose metabolism (e.g., oral or parenteral steroids)
(5) Active treatment for or history of a major medical illness such as coronary heart disease
(6) Previous formal training in meditation and other mind/body practices including yoga, tai chi, or qi gong
(7) Psychosis or significant depression, anxiety, or substance abuse under active care (>2 mental healthcare visits per month) or requiring more than 2 psychotropic medicines daily or hospitalization within the past 2 years
(8) Pregnancy or anticipated pregnancy
(9) Impaired cognition (inability to follow and respond appropriately during screening)
(10) Lack of self-reported life stress

^a^Participants were asked “How would you describe your general level of stress when you consider the multiple areas of your life?” This includes, but is not limited to your work life, your family or social life and your financial situation.” Response scale included 0 = *no stress at all*, 1 = *some stress*, 2 = *quite a bit of stress*, and 3 = *extreme stress*.

**Table 3:** Six-month postintervention interview questions.

#	Question
1	Describe how you have followed up with the practices you learned in the eight-week program you attended.
2	What practices or tools that you learned during the program were most helpful in continuing to work towards preventing diabetes?
3	What if any barriers did you find in continuing to work towards preventing diabetes?
4	Did you participate in the booster sessions?
4(a)	If so, what did you find most helpful, and what suggestions for improvement can you give us?
4(b)	If not, what were the barriers or other reasons for not participating in the booster sessions?
5	Reflecting back on the program and the follow-up period, what suggestions do you have for improvement in all aspects of the program?

**Table 4:** Characteristics of the study population.

Variable	Categories	Control *N* (SD%) (*N* = 38)	Treatment *N* (SD%) (*N* = 30)
Age		52.66 (9.94)	52.48 (9.55)
Sex	Female	28 (73.7%)	18 (60.0%)
	Male	4 (10.5%)	6 (20.0%)
Relationship	Single	11 (28.9%)	6 (20.0%)
	Married or committed relationship	17 (44.6%)	17 (56.6%)
	Divorced/separated/widowed	5 (13.1%)	3 (9.9%)
Highest level of education	Less than high school completion	0 (0.0%)	0 (0.0%)
	Graduated from high school or have GED	4 (10.5%)	4 (13.3%)
	Some college or associates/technical degree	12 (31.4%)	6 (20.0%)
	College degree or greater	16 (42%)	15 (49.9%)
Insurance	Medicare/medicaid	8 (20.9%)	5 (16.6%)
	Private insurance	18 (47.3%)	16 (53.3%)
	Self-pay, uninsured	4 (10.5%)	6 (20.0%)
	Others	8 (21.0%)	2 (6.6%)
Have a religious affiliation and/or spiritual practice	Yes	31 (81.5%)	24 (80.0%)
	No	1 (2.6%)	1 (3.3%)
Employment	Caretaker/homemaker/retired	13 (34.2%)	9 (29.9%)
	Current student	1 (2.6%)	1 (3.3%)
	Disabled	4 (10.5%)	1 (3.3%)
	Working outside the home	13 (34.2%)	10 (33.3%)
	Unemployed at present	3 (7.8%)	3 (10.0%)
Income	Less than $40,000	13 (34.1%)	9 (30.0%)
	$41,000−$80,000	7 (18.3%)	11 (36.7%)
	More than $80,000	9 (23.6%)	3 (10.0%)

**Table 5:** Credibility of the interventions at 2 weeks and 7 weeks.

	MDP (*N* = 38)	CDP (*N* = 30)	Between-group difference
	Mean (SD)	Mean (SD)	
Logical			
2 weeks	7.04 (1.93)	7.71 (1.08)	
7 weeks	7.26 (1.56)	8.35 (0.78)	
Within-group change	−0.03 (1.33)	0.47 (0.94)	0.50
Confident			
2 weeks	6.86 (1.96)	7.71 (1.27)	
7 weeks	7.32 (1.42)	8.00 (1.21)	
Within-group change	0.17 (1.26)	0.20 (0.76)	0.03
Recommend			
2 weeks	7.04 (2.17)	8.14 (1.21)	
7 weeks	7.63 (1.26)	8.57 (0.73)	
Within-group change	0.43 (1.41)	0.40 (0.97)	−0.03
Important			
2 weeks	7.61 (1.97)	8.50 (0.88)	
7 weeks	8.37 (0.68)	8.65 (0.57)	
Within-group change	0.40 (1.16)	0.40 (1.16)	−0.27
Successful			
2 weeks	7.43 (1.79)	7.50 (1.53)	
7 weeks	8.05 (0.91)	7.91 (1.53)	
Within-group change	0.37 (1.16)	0.37 (1.03)	−0.57

Credibility was assessed at the second week and the seventh week intervention sessions. The response options ranged from 0 = *not at all* to 9 = *very.* Logical: How logical does this type of treatment seem to you? Confident: How confident are you that this treatment would be successful in helping you prevent diabetes? Recommend: How confident would you be in recommending this treatment to a friend who wanted to prevent diabetes? Important: How important do you think it is that we make this treatment available to others who want to prevent diabetes? Successful: How successful do you believe this treatment would be in decreasing other problems involving tension, anxiety, insomnia, etc.?

**Table 6:** Means and with-in group changes by group and time-point (HOMA-IR, A1C, BMI, waist-hip ratio, perceived stress, cortisol, 7-day physical activity, dietary variables, FACIT-SP-EX)^a^.

Variables	Time	Treatment (*n* = 38)		Control (*n* = 30)	
		Mean (SD)/median	Within-group change from baseline (95% CI)	Mean (SD)/median	Within-group change from baseline (95% CI)
HOMA-IR	Baseline	4.88 (7.25)/2.19^b^		6.82 (9.85)/3.36	
	2 weeks	5.22 (6.90)/2.56	0.35 (−2.51, 3.20)	6.00 (7.55)/3.40	−0.82 (−5.22, 3.58)
	3 months	5.59 (9.12)/2.65	0.71 (−2.17, 3.60)	8.69 (12.74)/3.27	1.87 (−4.00, 7.74)
	6 months	6.63 (10.08)/2.90	1.76 (−1.90, 5.42)	5.31 (6.36)/2.96	−1.51 (−5.50, 2.49)
A1C (%) (mmol/mol)	Baseline	5.98 (0.41)		6.14 (0.32) [43]	
	3 months	5.89 (0.40)	−0.08 (−0.13, −0.03)	6.03 (0.34) [41]	−0.11 (−0.19, −0.03)
	6 months	5.85 (0.46)	−0.12 (−0.19, −0.06)	5.93 (0.29) [40]	−0.21 (−0.29, −0.14)
Body mass index (BMI)	Baseline	34.89 (6.54)		36.86 (6.21)	
	2 weeks	34.57 (6.58)	(−0.15) (−1.07, 0.40)	36.58 (6.44)	(−0.28) (−0.65, 0.27)
	3 months	34.54 (6.51)	(−0.35) (−1.15, −0.11)	36.37 (6.39)	(−0.49) (−1.69, 0.33)
	6 months	34.44 (6.32)	(−0.45) (−1.14, 0.05)	36.14 (6.36)	(−0.72) (−2.70, 0.10)
Wait-hip ratio	Baseline	0.43 (0.07)		0.44 (0.04)	
	2 weeks	0.43 (0.07)	0.001 (−0.004, 0.005)	0.44 (0.04)	0.001 (−0.005, 0.006)
	3 months	0.43 (0.07)	0.002 (−0.003, 0.007)	0.45 (0.04)	0.004 (−0.003, 0.01)
	6 months	0.43 (0.07)	0.001 (−0.004, 0.005)	0.45 (0.04)	0.002 (−0.004, 0.008)
Perceived stress (0−16 scale)	Baseline	4.35 (3.57)		4.07 (3.18)	
	2 weeks	3.93 (3.29)	(0.82 (−1.74, 0.10)	4.66 (2.87)	0.41 (−0.65, 1.47)
	3 months	3.76 (3.54)	−1.07 (−2.22, −0.09)	4.62 (3.07)	0.50 (−0.76, 1.76)
	6 months	4.31 (3.27)	−0.27 (−1.48, 0.94)	4.25 (3.08)	0.00 (−1.39, 1.39)
Cortisol	Baseline	0.27 (0.51)		0.16 (0.10)	
	2 weeks	0.24 (0.48)	−0.04 (−0.12, 0.05)	0.14 (0.06)	−0.02 (−0.07, 0.02)
	3 months	0.15 (0.07)	−0.12 (−0.29, 0.05)	0.13 (0.06)	−0.03 (−0.07, 0.02)
	6 months	0.14 (0.07)	−0.13 (−0.30, 0.04)	0.14 (0.06)	−0.02 (−0.07, 0.03)
Daily calorie expenditure (physical activity)	Baseline	3253 (671)		3603 (672)	
	3 months	3204 (643)	−48.6 (−153, 56.1)	3596 (732)	−7.7 (−105, 89.6)
	6 months	3220 (693)	−33.4 (−110, 42.9))	3589 (740)	−14.6 (−153, 124)
Daily calorie intake	Baseline	1940 (1448)		1826 (740)	
	3 months	1655 (1277)	−285 (−490, −80)	1695 (686)	−131 (−368, 106)
	6 months	1575 (1300)	−365 (−554, −176)	1509 (743)	−316 (−646, 14)
Daily fat (g)	Baseline	88 (71)		82 (31)	
	3 months	76 (67)	−13 (−21, 4.7)	78 (35)	−4.7 (−17, 7.6)
	6 months	72 (68.97)	−16 (−25, −7.1)	67 (37)	−16 (−32, 1.0)
Daily carbohydrate (g)	Baseline	219 (161)		203 (109)	
	3 months	181 (132)	−37 (−67, −7)	185 (74)	−18 (−49, 13)
	6 months	178(136)	−41 (−62, −19)	171 (84)	−32 (−75, 12)
FACIT-23-item total	Baseline	72.96 (11.94)		73.37 (8.69)	
	2 weeks	74.27 (8.64)	2.66 (−0.57, 8.43)	72.72 (11.39)	−0.46 (−5.09, 3.09)
	3 months	75.29 (8.43)	2.93 (−0.57, 8.57)	71.64 (11.95)	−0.33 (−5.43, 3.85)
	6 months	74.85 (9.52)	1.96 (−9.13, −0.57)	73.71 (8.02)	0.77 (−7.96, −1.78)

^a^The data at the time point after two weeks were not collected for A1C, physical activity, daily calories, daily fat, and daily carbohydrate. ^b^Medians are included for variables with skewed distributions. Abbreviations: HOMA-IR = homeostatic model assessment of insulin resistance; FACIT Sp Ex = expanded functional assessment of chronic illness therapy-spiritual well-being.

**Table 7:** Key intervention design modifications for a future, definitive RCT.

Participant qualitative feedback	Intervention design modifications^*∗*^
*Timing of intervention sessions*	

Participants shared feedback to guide scheduling, including consideration of life demands, familial obligations, and weekend and evening sessions. Mixed information was shared, including having longer sessions with fewer total session days. In contrast to those who suggested fewer session days, some participants requested more face-to-face sessions, twice-monthly booster sessions, additional half-day sessions, and a longer follow-up period beyond six months. Others verbalized appreciation for having sessions at a school in the target community versus at the medical center.	Eight sessions will be held one evening per week, every other week, and across sixteen weeks. Participants who have to miss a session will have the opportunity to make up that session during the week that class is not scheduled. Session one will include intervention orientation components designed to promote equitable and high credibility among both intervention arms. One, half-day Saturday retreat will be held. After the 8 weekly sessions, once-per-month booster sessions take place, with the availability of make-up sessions. This adds flexibility to the intervention design and accounts for the likelihood that life obligations may cause participants to miss sessions. Intervention content will include mindfulness strategies to help participants integrate and sustain self-care/health-promoting behaviors in the context of demands and caregiving obligations. Because the eight intervention sessions will be implemented across 16 weeks, followed by 6 months of booster sessions, participants will be engaged in the study's intervention activities during a time span that is two months longer than the feasibility study. During recruitment of each cohort, participants will receive a query about familial obligations during weeknights and Saturdays. This information will be used to schedule interventions sessions on days that are most feasible for the majority of participants.

*Location of intervention sessions and data collection*	
Some participants expressed the potential benefits of having sessions at a more centrally located community or church setting. Some also shared challenges to their attendance at the UNC CTRC hospital-based laboratory data collection visits including traffic, parking, and medical center location. Individuals' suggestions for overcoming these challenges included having nighttime appointments and an off-site location, such as a local community clinic.	Data collection and intervention sessions will be held at community locations that are conveniently located, with adequate parking, and adjacent to local bus stops. Community locations will have private areas for lab testing, survey data collection, and space for health education and exercise sessions.

*Implementation of intervention strategies at home*	
Participants reported challenges with “making time” to meditate and do the assigned homework. Some noted challenges with balancing work and family obligations to meditate consistently, cook, and eat healthier foods and integrate physical activity into their lives. Individual participants made suggestions regarding the need for increased group and peer support, enhanced accountability, access to study-specific videos to enhance exercise, and tips for organizing time to incorporate exercise and healthy behavioral change.	Web-accessible exercise and mindfulness videos, a mindfulness app to support home practice, a phone app for nutrition monitoring, onsite childcare, reminder phone calls, and a peer support “buddy system” will be implemented to provide encouragement and support to participants as they incorporate/maintain healthy behaviors into their routines.

^*∗*^The investigators will work with a community advisory board during the design and implementation of a larger definitive RCT to maximize cultural relevance, acceptability, outreach/recruitment, impact, and future translation and dissemination of the project.

## Data Availability

Deidentified data used to support the findings of this study are available from the corresponding author upon request and approval of the Institutional Review Board.
